# The “chameleon” sellar lesions: a case report of unexpected sellar lesions

**DOI:** 10.3389/fneur.2023.1149858

**Published:** 2023-04-24

**Authors:** Ilaria Bove, Raduan Ahmed Franca, Lorenzo Ugga, Domenico Solari, Andrea Elefante, Maria Laura Del Basso De Caro, Luigi Maria Cavallo

**Affiliations:** ^1^Division of Neurosurgery, Department of Neurosciences, Reproductive and Odontostomatological Sciences, Università degli Studi di Napoli “Federico II”, Naples, Italy; ^2^Department of Advanced Biomedical Sciences, Pathology Section, University of Naples “Federico II”, Naples, Italy; ^3^Department of Advanced Biomedical Sciences, University of Naples “Federico II”, Naples, Italy

**Keywords:** sellar region, oncology, neuroimaging, endoscopic endonasal surgery, pathology

## Abstract

**Introduction:**

The sellar region and its boundaries represent a challenging area, harboring a variety of tissues of different linings. Therefore, a variety of diseases can arise or involve in this area (i.e., neoplastic or not). A total of three challenging cases of “chameleon” sellar lesions treated via EEA were described, and the lesions mimicked radiological features of common sellar masses such as craniopharyngiomas and/or pituitary adenomas, and we also report a literature review of similar cases.

**Methods:**

A retrospective analysis of three primary cases was conducted at the Università degli Studi di Napoli Federico II, Naples, Italy. Clinical information, radiological examinations, and pathology reports were illustrated.

**Results:**

A total of three cases of so-called “chameleon” sellar lesions comprising two men and one woman were reported. Based on the intraoperative finding and pathological examination, we noticed that case 1 had suprasellar glioblastoma, case 2 had a primary neuroendocrine tumor, and case 3 had cavernous malformation.

**Conclusion:**

Neurosurgeons should consider “unexpected” lesions of the sellar/suprasellar region in the preoperative differential diagnosis. A multidisciplinary approach with the collaboration of neurosurgeons, neuroradiologists, and pathologists plays a fundamental role. The recognition of unusual sellar lesions can help surgeons with better preoperative planning; so an endoscopic endonasal approach may represent a valid surgical technique to obtain decompression of the optic apparatus and vascular structures and finally a pathological diagnosis.

## 1. Introduction

The sellar and the suprasellar regions represent a very complex area, harboring a remarkable variety of tissues of different linings, and many diseases can arise from or involve these areas, with a majority of them from hypophysis, both neoplastic or not (1). Over 90% of sellar tumors are pituitary adenomas that are recently redefined as pituitary neuroendocrine tumors (PitNETs) to underline their unpredictable behavior (2, 3). In nearly 10% of cases, other etiologies are responsible for the mass effect in the sellar region including gliomas, meningiomas, craniopharyngiomas, and Rathke's cysts, and vascular lesions like aneurysms and cavernous angiomas may also be rarely encountered in the sellar region (1). Rapid recognition of the sellar masses is crucial to determine prognostic outcomes and therefore guide management (4). Over the past century, we have assisted a vivid development of endoscopic skull base surgery, along with advances in diagnostic imaging techniques: The endoscopic endonasal approach allows access to the multiple and various lesions of the sellar–suprasellar areas that were previously accessible only via the transcranial routes (5). The main advantage of the endoscopic endonasal approach (EEA) lies in the possibility of obtaining a close-up view of the neurovascular structures, reducing overall tissue manipulation (6–8). The most common lesions arising from this region have a distinctive radiological appearance; however, in some cases, “unexpected” masses may mimic the radiological characteristics typical of common sellar pathologies. Neuroradiological detection of complex sellar–suprasellar lesions can sometimes be extremely difficult. In recent years, the advancement of neuroimaging has been investigated in order to provide information to improve diagnostic accuracy, including a description of tumor cell biology, cerebral blood perfusion, and vascular proliferation characteristics. In this context, radiomics has become an interesting and continuously evolving technique. It represents a tool capable of building decision support models based on conventional or functional imaging, thanks to the extraction of large quantities of image features and quantitative data analysis (9).

Herein, we report three challenging cases of “chameleon” sellar lesions treated via EEA that mimicked radiological features of common lesions such as craniopharyngiomas and/or pituitary adenomas, with the literature review of similar cases.

## 2. Illustrative cases

### 2.1. Case 1. Suprasellar glioblastoma

**Figure 1:**
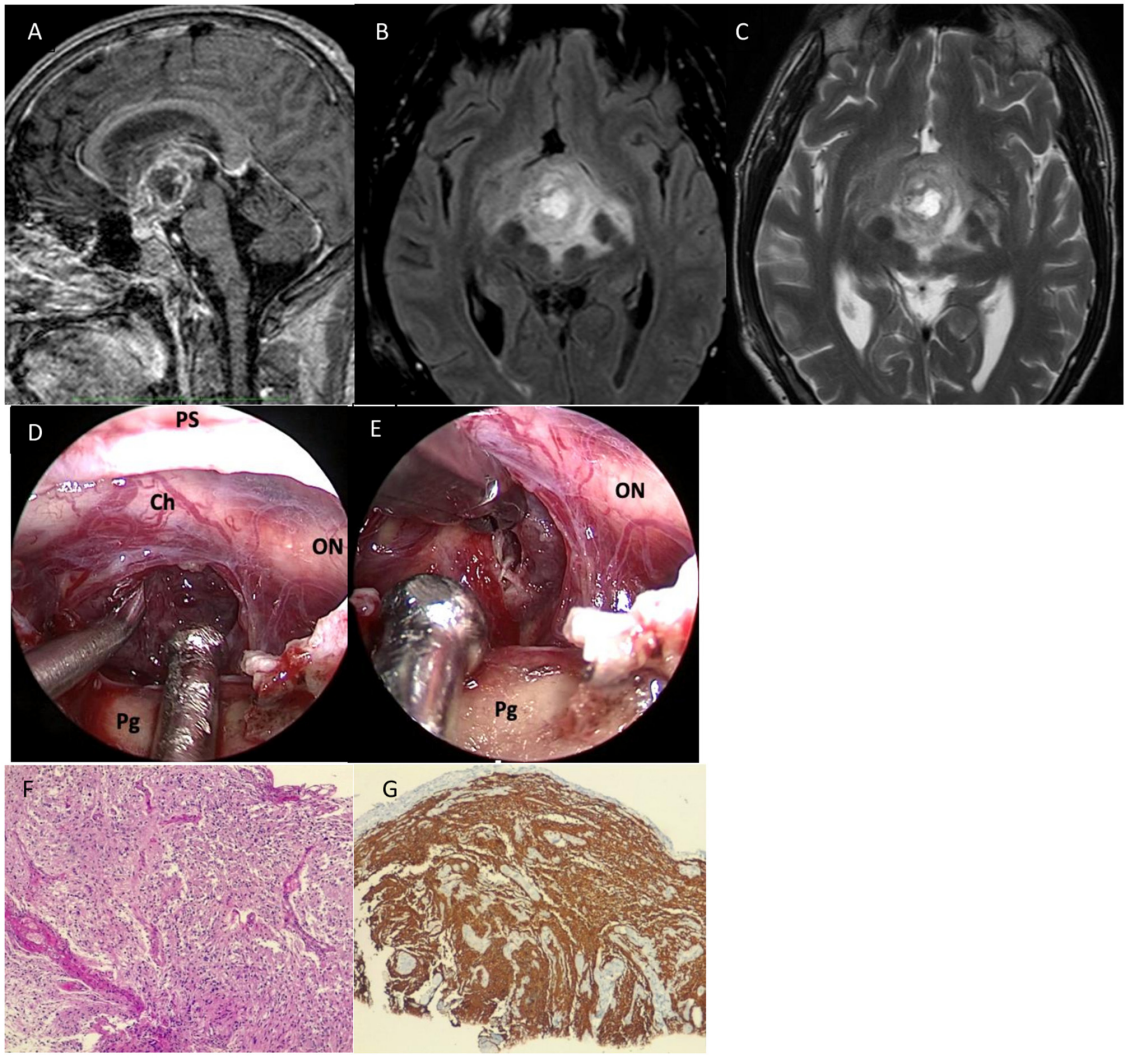
Preoperative sagittal **(A)**, axial **(B)** T2, and axial **(C)** T2-fluid-attenuated inversion recovery (FLAIR) sequences showed a huge mass located in the median and paramedian portion of the hypothalamus-chiasmatic region (Dmax 50 cm), with irregular margins infiltrating the uncus-amygdaloid complex in the hypothalamus region and the floor of the third ventricle; the lesion presented central colliquative necrotic component and its signal was relatively homogenous with slightly hypointense in T1 and hyperintense on T2-weighted images, with significant heterogenous post-Gad enhancement and peripherical vasogenic edema. **(D)** After dural opening, the lesion appeared diffusely infiltrating the infundibulum and third ventricle, and it presented as a grayish-yellow tissue and was highly vascularized. **(E)** Further resection of the tumor was not performed because the tumor adhered tenaciously to surrounding structures. **(F)** Histological examination revealed a highly cellular neoplasm having a fibrillary background, composed of pleomorphic, medium-sized cells. Necrosis and microvascular proliferation were also seen (*hematoxylin-eosin, original magnification 10x*). **(G)** On immunohistochemistry, tumor cells were GFAP positive (*immunoperoxidase staining, original magnification 10x*). IDH1 immunostaining was negative (not shown in the figure). ON, optic nerve; Ch, chiasm; T, tumor; Pg, pituitary gland.

**Figure 2:**
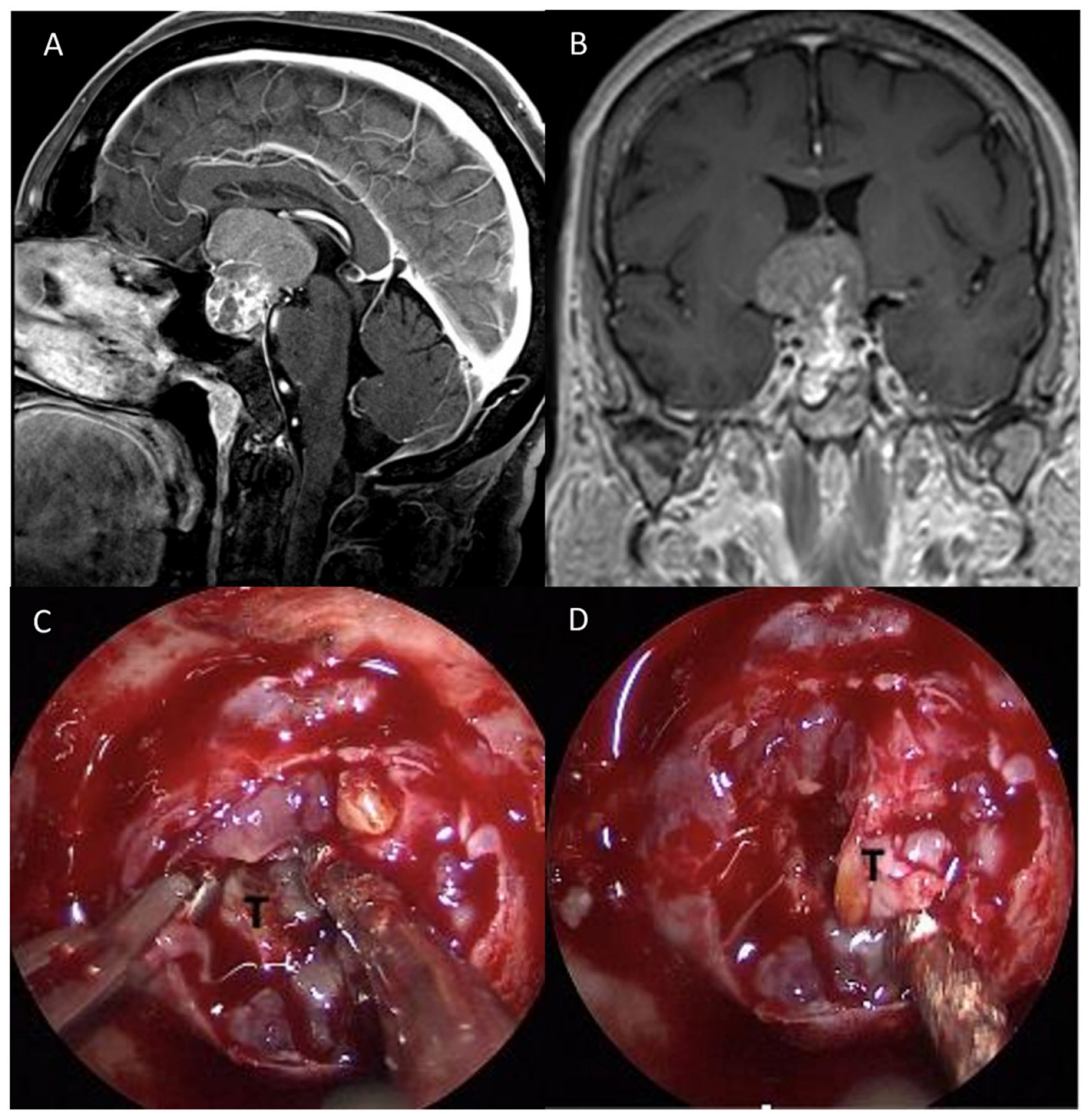
Preoperative sagittal **(A)** and coronal **(B)**, post-gadolinium MRI scan showing a suprasellar mass (Dmax 3.9 cm) with the third ventricle involvement and heterogenous contrast enhancement compressing the optic chiasm. **(C)** After dural opening, **(D)** a fibro-elastic and infiltrating lesion was partially removed in order to obtain optic nerve decompression.

A 46-year-old man was admitted to our department with a frontal headache and vomiting. Bitemporal hemianopsia, spatial–temporal disorientation, and memory loss were detected upon hospital admission. Laboratory examination revealed an increase in the level of PIVKA (75 n.v.16–48 AU/mL). In 1996 and later in 2006, the patient underwent left and then right orchidectomy for testicular seminoma. Brain MRI showed a huge mass located in the median and paramedian portion of the hypothalamus–chiasmatic region (Dmax 50 cm), with irregular margins infiltrating the uncus–amygdaloid complex in the hypothalamus region and the floor of the third ventricle; the lesion presented a central colliquative necrotic component, and its signal was relatively homogenous with slightly hypointense images in T1 and hyperintense on T2-weighted images, with significant heterogenous post-Gad enhancement. Signs of supratentorial hydrocephalus were noticed. The first radiological impression was compatible with a case of craniopharyngioma. An extended suprasellar endoscopic endonasal approach was run for tumor removal: Upon dural opening, the lesion appeared diffusely infiltrating the infundibulum and third ventricle, and it presented as a grayish-yellow tissue and was highly vascularized (**Figure 2**). Intraoperative histological examination revealed the presence of a malignant glial cell tumor. Further resection of the tumor was not performed because the tumor adhered tenaciously to surrounding structures. During the postoperative course, due to the presence of supratentorial hydrocephalus, a biventricular peritoneal shunt with a 130 cm H_2_O Codman programmable valve was positioned in a second surgery. A path report revealed an IDH1-wild-type glioblastoma. Adjuvant radio and concomitant chemotherapy treatment were started immediately as per the STUPP protocol. The patient died 1 year after the surgery due to the progression of the disease (Figure 1).

### 2.2. Case 2. Sellar primary neuroendocrine tumor

**Figure 3:**
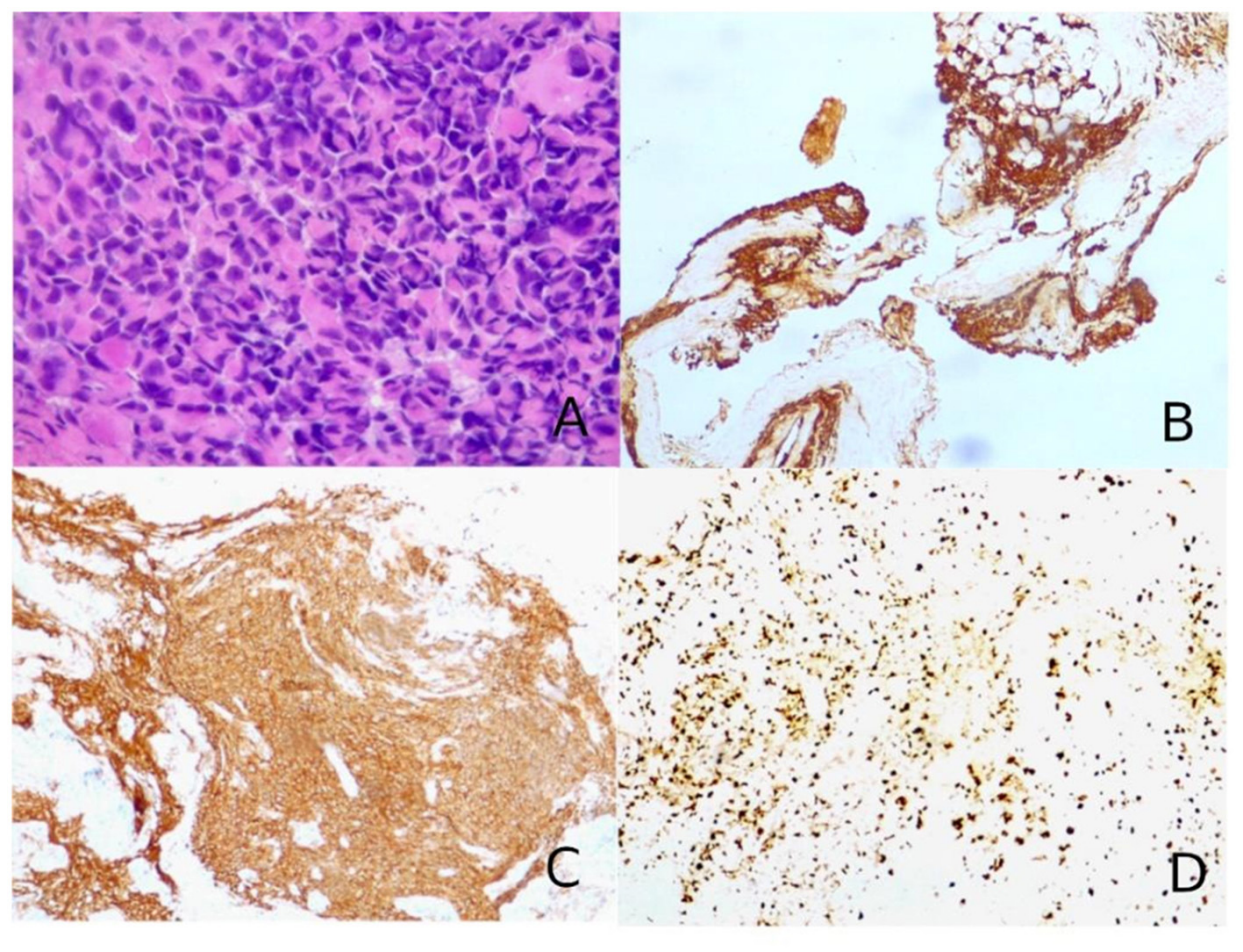
**(A)** Hematoxylin–eosin slides showed a hypercellular tumor composed of large, epithelioid, often nucleolated cells, with abundant cytoplasm, arranged in a “vertebral-like” fashion (*hematoxylin–eosin, original magnification 40x*). **(B)** Tumor cells were positive for pancytokeratin (AE1/AE3; *immunoperoxidase staining, original magnification 10x*). **(C)** Neuroendocrine markers were consistently positive, as for synaptophysin shown in the figure (*immunoperoxidase staining, original magnification 10x*). **(D)** The cellular proliferation index Ki67 was nearly 70–80% (*immunoperoxidase staining, original magnification 10x*).

A 50-year-old woman with a history of cervix adenocarcinoma was admitted to our department with a headache and visual disturbance. MRI with enhancement post-contrastographic revealed the presence of suprasellar mass (Dmax 3.9 cm) with the third ventricle involvement with heterogenous contrast enhancement compressing the optic chiasm. A *transtuberculum/transplanum* endoscopic endonasal approach was performed. A fibro-elastic and infiltrating lesion was partially removed in order to obtain optic nerve decompression. Intraoperative histological examination showed the presence of atypical cells with a plasmacytoid appearance (Figure 2). The lesion appeared very firmly adherent to surrounding structures, so, after decompression of the optic chiasm, a small residual mass of the tumor was left in place. Histology and immunohistochemical staining ultimately confirmed the diagnosis of a primary neuroendocrine tumor (Figure 3). Laboratory examination revealed an increase in the level of neuron-specific enolase NSE (68.7 mcg/L; v.n. <18.3). During the postoperative course, the patient reported an improvement in visual acuity. Follow-up ^18^F-FDG-PET/CT revealed the absence of any localization of the disease, while MRI showed the decompression of the optic apparatus despite a large intra-suprasellar residual lesion.

### 2.3. Case 3. Suprasellar cavernous malformations

A 21-year-old man was admitted to our department with a headache and sudden visual loss. Ophthalmological examination revealed 1/30 in RE with diffuse reduction of light sensitivity and bitemporal hemianopia in LE. Endocrinological assessment and lab essays revealed central hypercortisolism. Brain computed tomography imaging demonstrated hyperdense large sellar and suprasellar mass with extension into the third ventricle cavity with the presence of calcifications. MRI showed a heterogenous low signal in T1 images, an intermediate high signal in T2 images, and cystic with calcific components of the suprasellar lesion. It measured ~3 × 2, 4 × 3, and 3 cm in anteroposterior, cephalocaudal, and transverse dimensions, respectively.

**Figure 4:**
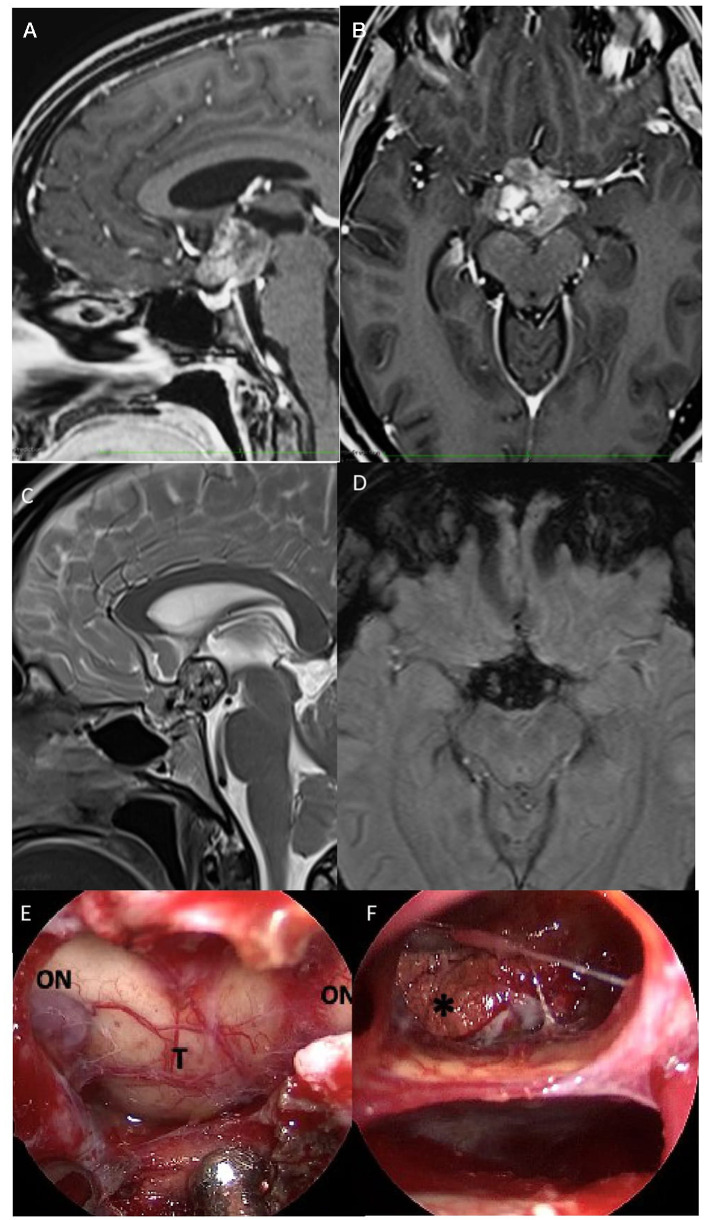
Preoperative sagittal **(A)** axial **(B)** post-gadolinium, and sagittal **(C)** T2 MRI scan demonstrated a suprasellar mass with heterogenous low signal in T1 images and an intermediate high signal in T2 images, with cystic and calcific components of the lesion. Axial **(D)** susceptibility-weighted imaging (SWI) showed signal dropout (hypointensity) in the sellar region. The lesion measured ~3 × 2,4 × 3, and 3 cm on anteroposterior, cephalocaudal, and transverse dimensions. **(E)** After dura opening, the evacuation of the intralesional hemorrhagic component localized inside the optic chiasm and infundibulum of the third ventricle was performed, which appeared dislocated below; **(F)** at the end of the procedure, a yellowish granulomatous formation was removed in fragments of dubious vascularization but suspected of a possible, already site of previous bleeding and adhering to the ventricular walls. ON, optic nerve; T, tumor. *Calcification.

An extended endoscopic endonasal approach was performed. During surgery, the evacuation of the intralesional blood component of the neoformation localized inside the optic chiasm and infundibulum of the third ventricle was performed, which appeared dislocated below; at the end of the procedure a yellowish granulomatous formation was removed in fragments, of dubious vascularization, but suspected of a possible, already site of previous bleeding and adhering to the ventricular walls. Considering its high vascularity and the difficulty of dissection from the adjacent structures, a subtotal resection of the lesion aiming at optic nerve decompression was achieved (Figure 4). Histopathological examination was consistent with cavernous malformation.

The patient's visual acuity improved on postoperative day 3, and MRI showed decompression of the optic apparatus despite a large intra-suprasellar residual lesion. A second-stage surgery was proposed to the patient to obtain a more radical excision, but he refused. The neuro-oncological multidisciplinary team meeting discussed the case, and considering the patient's decision, the patient underwent stereotactic radiosurgery. At the follow-up visit after 6 and 12 months, the residual lesion is stable, and the patient did not develop any new neurological signs maintaining the visual improvement.

## 3. Discussion

Tumors of the sellar region account for ~10–15% of all brain tumors, and a large variety of non-neoplastic, inflammatory, vascular, or developmental lesions can be found in this region (10, 11).

Pituitary adenomas constitute over 90% of sellar masses, while the remaining 10% of the lesions includes pituitary-origin tumors, such as craniopharyngiomas, Rathke's cleft cysts, and astrocytomas, and non-pituitary origin lesions, such as meningiomas, germ cell tumors, chondrosarcomas/chordomas, giant cell tumors, epidermoid cysts, and metastatic lesions (12).

According to previous data, rare sellar lesions represent a heterogeneous group of non-adenomatous lesions that deserve special care regarding their surgical and clinical management (1, 13). A total of 2,452 consecutive patients were operated on via an endoscopic endonasal approach for the removal of a sellar/parasellar lesion at the Division of Neurosurgery of the Università degli Studi di Napoli Federico II, Naples, between January 1997 and January 2023; of them, a total of 118 rare sellar lesions were identified (4.8%). Somma et al. (1) affirmed how several signs (i.e., DI and ophthalmoplegia) and neuroradiological features (i.e., intense and homogeneous contrast enhancement, invasive aspect of the lesion) should induce suspicion of non-adenomatous diseases. In the three cases reported, the suspicion of rare/unexpected sellar lesions was low due to the non-pathognomonic clinical presentation and radiological appearance.

Brain MRI is routinely adopted for diagnosis and proper identification of sellar lesions details and features; however, MRI appearance of different sellar/parasellar lesions can be very similar though misleading (14, 15). The differentiation between several tumor types based on radiological features can sometimes be difficult on conventional radiological examinations because of the overlapping MRI findings (16). Based on recent studies, MRI imaging can provide information on the consistency of macroadenomas, craniopharyngiomas, and germ cell tumors (17); Khant et al. (18) demonstrated how the TSE-ADC images may aid to differentiate craniopharyngioma from pituitary adenomas, and DWI sequences should distinguish craniopharyngiomas from germ cell tumors. Other imaging modalities, such as somatostatin receptor scintigraphy, can help in the differential diagnosis (19). Although the presence of unusual sellar masses is rare, suspicion should always be based on the history, clinical presentation, and radiological appearance. The diagnostic workup and management in these cases should require a specialized multidisciplinary team including neurosurgeons, neuroradiologists, endocrinologists, oncologists, and pathologists.

### 3.1. Glioma

Glioblastoma (GBM) is the most common adult brain tumor, occurring in the subcortical white matter of the cerebral hemispheres (20). From the literature review, only five cases of sellar and suprasellar GBM have been reported. In all cases, sellar GBM mimicked common sellar lesions, such as pituitary macroadenoma and/or craniopharyngioma; four cases underwent surgery via endoscopic endonasal surgery, and two cases underwent transcranial surgeries. Lemm et al. (21) reported two cases with preoperative suspect of craniopharyngiomas; Mahta et al. (22) and Anvari et al. (23) reported in both cases the preoperative workup pointed toward the suspicion of a pituitary macroadenoma. The case reported by Deng et al. (24) was a 42-year-old woman with an intra- and suprasellar not well-defined lesion, presenting headache, amenorrhea, diabetes insipidus, visual loss, and visual field defect. In five of six cases, including our case (Case 1), the sellar GBM originated from the hypothalamic/pituitary axis and from the pituitary gland. Thus, the onset symptoms were endocrinological abnormalities and visual and cognitive disturbances.

Sellar and/or suprasellar gliomas are usually low-grade glioma, i.e., optic nerve pilocytic astrocytoma associated with neurofibromatosis NF-1 (25). Appearance on MRI may vary; glial lesions can appear hypodense or isodense and, in some cases, hyperdense. The presence of calcifications is rare; in these cases, the lesion appears isointense on T1 and lacks a cystic component (26). The rarity of malignant gliomas lies in uncommon localization in this region and in their heterogeneous presentation on neuroimaging, making this diagnosis very challenging before obtaining the tissue for histological analysis. Radiographically, craniopharyngioma is characterized by heterogeneous solid tissue, cystic regions, and calcification. In T2-weighted images, the cysts are predominantly hyperintense, and the solid components present a heterogeneous signal. Post-contrast, there is a heterogeneous increase in contrast of the solid portions, as well as of the walls of the cysts (27). The presence of protein, cholesterol, and/or methemoglobin may determine a high signal, which would be more likely encountered in craniopharyngioma.

Albeit gadolinium enhancement MRI is observed in both craniopharyngioma and high-grade gliomas, the latter are less likely to present cystic degeneration and calcifications (28). In the differential diagnosis of lesions with central necrosis, the presence of a brain abscess is also included. In this case, correlation with clinical status, i.e., the presence of infectious signs like fever and increased inflammatory indices together with diffusion-weighted imaging (DWi), with diffusion restriction in the abscess, may help in the differential diagnosis between these two rare entities.

### 3.2. Primary neuroendocrine tumor

Neuroendocrine tumor (NET) arises from the neoplastic transformation of enterochromaffin cells (29). These epithelial cells are usually found in all human organs, especially in the gastrointestinal tract and respiratory system (29, 30). According to the recent literature, two cases of sellar primary intracranial NET have been reported (31, 32). Liu et al. (31) reported a case of sellar/suprasellar NET. This is a case of a patient who underwent a single nostril transsphenoidal approach to obtain a gross tumor removal. The pathological diagnosis revealed the presence of high-grade small cell NET, and the patient died after 3 months of extensive metastases. Nasi et al. (32) reported a successful case; after a subtotal resection by an endoscopic endonasal approach, the patient underwent fractioned stereotactic radiotherapy (total irradiation dose 43.1 Gy) and polychemotherapy (cisplatin, ifosfamide, and etoposide). Four years later, the follow-up MRI showed a stable residual disease without any neurological complications.

Usually, patients with NET do not have specific clinical features, but in presence of functional tumors, they may develop endocrine symptoms from secreting one or more hormones, while non-functional tumors may affect pituitary gland function leading hypopituitarism (33). In our case, the lesion invaded the suprasellar region with compression of the optic chiasm and third ventricle involvement, without endocrine dysfunction.

CT and MRI are not specific radiological investigations for these tumors; as demonstrated by our case, NET presents MRI findings similar and compatible with other more common pathologies of the sellar region, such as pituitary adenoma, meningioma, and metastases (34). It is known as NETs express somatostatin receptor subtypes type 2 and 5; therefore, in these cases, it would be useful for diagnostic purposes to perform a somatostatin receptor scintigraphy. Scintigraphy may aid in the differential diagnosis, as well as staging and monitoring of this tumor (31, 33). It has been shown that a PET scan with 11C-5-hydroxytryptophan can be effective in tracing small NETs, with a significantly higher detection rate than somatostatin receptor scintigraphy in most cases (30, 31, 34). On the contrary, since NET is characterized by low cellular proliferative activity and high differentiation rate, positron emission tomography scanning with 18F-labeled fluorodeoxyglucose is not a technique to detect this tumor (35).

### 3.3. Cavernous malformation

The cavernous malformation (CM) can affect any cerebral region, but it is more frequent as it tends to affect the subcortical areas of the frontal and temporal lobes, while in the posterior fossa, it tends to involve the pons and the cerebellar hemispheres, however, medulla involvement is uncommon (36). However, in rare cases, the sellar region is involved (37). Impaired vision and/or cranial nerve palsies are common clinical findings, but all of these manifestations cannot aid in differentiation since they are usually present in other common pathologies (e.g., pituitary adenomas, meningiomas, craniopharyngiomas, and Schwannomas) (38). However, in the literature, some MRI features have been reported that could raise the suspicion of CM of the sellar region. Indeed, the presence of a hyperintense signal in T2 sequences associated with delayed centripetal contrast enhancement on MRI images could raise suspicion (39). Due to the high vascularity and profuse bleeding of the lesion during surgical removal, a subtotal resection to obtain neurovascular decompression followed by radiotherapy might be considered the most effective strategy of treatment.

To date, only 16 operated cases were reported in the current literature, and total resection was achieved in two cases (37, 40–44). Multiple surgical approaches have reportedly been utilized including pterional and subfrontal craniotomies or sublabial, transseptal, and endoscopic endonasal transsphenoidal approaches.

Maximally safe resection should be performed, including decompression of the optic apparatus and cavernous sinus. Therefore, considering the nature of the lesion and its anatomical extension, an endoscopic endonasal approach (EEA), which allows easier access and feasible debulking of sellar masses, is advocated (38, 45–52). In case of preoperatively suspected and intraoperative confirmation of sellar CM via frozen section, partial resection should be attempted, paying particular attention to obtain complete hemostasis. Additional treatments, such as stereotaxic radiosurgery, should be considered for the management of the residual lesion, after histological confirmation, in order to avoid further morbidity (25). Radiation therapy has been used successfully both before and after surgery and is recently considered an effective treatment with an average 54% reduction in tumor volume (53). Given the excellent results of radiotherapy treatment and the low possibility of obtaining a total resection, surgery remains a controversial treatment modality, if not for biopsy.

## 4. Future perspectives

The recent advancement of the radiological technique is increasingly used by the surgeon, to plan the type of approach and the best treatment modality. Recent studies highlight how machine learning can provide additional information to support clinical decisions for neuroradiologists and neurosurgeons (40, 41). Histogram analysis, as part of quantitative plot analysis, evaluates the internal structure of tumors by analyzing the distribution of pixels or voxels in the image, which may not be visually perceptible to the human eye. Recent evidence suggests that it can be used to predict, for example, the histopathological and genomic characteristics of tumors and the response to treatment; it helps to evaluate the consistency and therefore be able to identify the tumor before histological evaluation (41). The possible clinical application of machine learning and radiomics of the sellar masses and other brain neoplasms, in general, should be adapted in a clinical model. Two systematic reviews performed by Saha et al. (54) and Qiao (55) summarize the application of machine learning in imaging analysis of the sellar lesion but only in pituitary adenomas. However, further research is necessary to understand the correct model that is most effective for the differential diagnosis and characterization of sellar lesions.

Neurosurgeons should consider the “unexpected” lesions of the sellar/suprasellar region in the preoperative differential diagnosis. The multidisciplinary approach with the collaboration of neurosurgeons, neuroradiologists, and pathologists plays a fundamental role. The proper diagnostic assessment of the sellar masses may help surgeons with better preoperative and postoperative planning, and in this scenario, the endonasal endoscopic approach could represent a fundamental surgical technique to obtain both a proper neurovascular structures decompression and a pathological diagnosis.

## 5. Conclusion

The presence of unusual sellar and suprasellar lesion features at the MRI associated with a rapidly worsening clinical course, altered hormonal profile, and cognitive disturbances should raise the suspicion of uncommon sellar lesions. From a radiological standpoint, the possibility of a malignant tumor diagnosis should be considered in case of evidence of invasion and infiltration of the surrounding tissues. Progress in imaging studies may help differentiate among the variety of possible lesions involving the suprasellar area. Further research and case series should be carried out in order to improve diagnosis and provide a proper strategy to ameliorate outcomes and ensure the overall survival of these patients.

## Data availability statement

The raw data supporting the conclusions of this article will be made available by the authors, without undue reservation.

## Ethics statement

Ethical review and approval was not required for the study on human participants in accordance with the local legislation and institutional requirements. The patients/participants provided their written informed consent to participate in this study. Written informed consent was obtained from the individual(s) for the publication of any potentially identifiable images or data included in this article. Written informed consent was obtained from the participant/patient(s) for the publication of this case report.

## Author contributions

IB: concept and design. IB, RF, and LU: data collection. IB and DS: original draft preparation. DS and AE: reviewing and editing. MD, DS, and LC: study supervision. All authors read and approved the final manuscript.

## Supplementary material

Supplementary Video 1 Intraoperative video of case 1 demonstrating an endoscopic endonasal approach for resection of suprasellar glioblastoma.

Supplementary Video 2 Intraoperative video of case 2 demonstrating an endoscopic endonasal approach for resection of sellar primary neuroendocrine tumor.

Supplementary Video 3 Intraoperative video of case 3 demonstrating an endoscopic endonasal approach for resection of suprasellar cavernous malformations.

## References

[1] Somma T Solari D Beer-Furlan A Guida L Otto B Prevedello D. Endoscopic endonasal management of rare sellar lesions: Clinical and surgical experience of 78 cases and review of the literature. World Neurosurg. (2017) 100:369–80. 10.1016/j.wneu.2016.11.057 27888088

[2] Asa SL Casar-Borota O Chanson P Delgrange E Earls P Ezzat S. From pituitary adenoma to pituitary neuroendocrine tumor (PitNET): An International Pituitary Pathology Club proposal. Endocr Relat Cancer. (2017) 24:C5–8. 10.1530/ERC-17-0004 28264912

[3] Asa SL Mete O Perry A Osamura RY. Overview of the 2022 WHO classification of pituitary tumors. Endocr Pathol. (2022) 33:6–26. 10.1007/s12022-022-09703-7 35291028

[4] Mete O Lopes MB. Overview of the 2017 WHO classification of pituitary tumors. Endocr Pathol. (2017) 28:228–43. 10.1007/s12022-017-9498-z 28766057

[5] de Divitiis E Cappabianca P Cavallo LM. Endoscopic transsphenoidal approach: Adaptability of the procedure to different sellar lesions. Neurosurgery. (2002) 51:699–705. 10.1097/00006123-200209000-00016 12188948

[6] Cappabianca P Cavallo LM Solari D Stagno V Esposito F de Angelis M. Endoscopic endonasal surgery for pituitary adenomas. World Neurosurg. (2014) 82:S3–11. 10.1016/j.wneu.2014.07.019 25496632

[7] Solari D Villa A De Angelis M Esposito F Cavallo LM Cappabianca P. Anatomy and surgery of the endoscopic endonasal approach to the skull base. Transl Med UniSa. (2012) 2:36–46. 23905043 PMC3728777

[8] Cavallo LM de Divitiis O Aydin S Messina A Esposito F Iaconetta G. Extended endoscopic endonasal transsphenoidal approach to the suprasellar area: Anatomic considerations–Part 1. Neurosurgery. (2008) 62(6Suppl.3):1202–12. 10.1227/01.NEU.0000333786.98596.33 18695541

[9] Koong K Preda V Jian A Liquet-Weiland B Di Ieva A. Application of artificial intelligence and radiomics in pituitary neuroendocrine and sellar tumors: A quantitative and qualitative synthesis. Neuroradiology. (2022) 64:647–68. 10.1007/s00234-021-02845-1 34839380

[10] Ezzat S Asa SL Couldwell WT Barr CE Dodge WE Vance ML. The prevalence of pituitary adenomas: A systematic review. Cancer. (2004) 101:613–9. 10.1002/cncr.20412 15274075

[11] Daly AF Rixhon M Adam C Dempegioti A Tichomirowa MA Beckers A. High prevalence of pituitary adenomas: A cross-sectional study in the province of Liege, Belgium. J Clin Endocrinol Metab. (2006) 91:4769–75. 10.1210/jc.2006-1668 16968795

[12] Fatemi N Dusick JR de Paiva Neto MA Kelly DF. The endonasal microscopic approach for pituitary adenomas and other parasellar tumors: A 10-year experience. Neurosurgery. (2008) 63(4Suppl.2):244–56. 10.1227/01.NEU.0000327025.03975.BA 18981830

[13] Cossu G Brouland J-P Rosa SL Camponovo C Viaroli E Daniel RT. Comprehensive evaluation of rare pituitary lesions: A single tertiary care pituitary center experience and review of the literature. Endocr Pathol. (2019) 30:219–36. 10.1007/s12022-019-09581-6 31209729

[14] Corsello SM Paragliola RM. Differential diagnosis of pituitary masses at magnetic resonance imaging. Endocrine. (2017) 58:1–2. 10.1007/s12020-017-1230-8 28092068

[15] Ugga L Franca RA Scaravilli A Solari D Cocozza S Tortora F. Neoplasms and tumor-like lesions of the sellar region: Imaging findings with correlation to pathology and 2021 WHO classification. Neuroradiology. (2023) 2023:1. 10.1007/s00234-023-03120-1 36799985 PMC10033642

[16] Kachhara R Nair S Gupta AK Radhakrishnan VV Bhattacharya RN. Infrasellar craniopharyngioma mimicking a clival chordoma: A case report. Neurol India. (2002) 50:198–200. 12134188

[17] Kinoshita Y Yamasaki F Tominaga A Ohtaki M Usui S Arita K. Diffusion-weighted imaging and the apparent diffusion coefficient on 3T MR imaging in the differentiation of craniopharyngiomas and germ cell tumors. Neurosurg Rev. (2016) 39:207–13. 10.1007/s10143-015-0660-0 26280640

[18] Khant ZA Azuma M Kadota Y Hattori Y Takeshima H Yokogami K. Evaluation of pituitary structures and lesions with turbo spin-echo diffusion-weighted imaging. J Neurol Sci. (2019) 405:116390. 10.1016/j.jns.2019.07.008 31476623

[19] Iglesias P Cardona J Díez JJ. The pituitary in nuclear medicine imaging. Eur J Intern Med. (2019) 68:6–12. 10.1016/j.ejim.2019.08.008 31519379

[20] van den Bent MJ Weller M Wen PY Kros JM Aldape K Chang S. clinical perspective on the 2016 WHO brain tumor classification and routine molecular diagnostics. Neuro Oncol. (2017) 19:614–24. 10.1093/neuonc/now277 28339700 PMC5464438

[21] Lemm D de Oliveira FH Bernays R-L Kockro RA Kollias S Fischer I. Rare suprasellar glioblastoma: Report of two cases and review of the literature. Brain Tumor Pathol. (2012) 29:216–20. 10.1007/s10014-012-0086-0 22350669

[22] Mahta A Buhl R Huang H Jansen O Kesari S Ulmer S. Sellar and supra-sellar glioblastoma masquerading as a pituitary macroadenoma. Neurol Sci. (2013) 34:605–7. 10.1007/s10072-012-1110-1 22569569

[23] Anvari K Samini F Faraji M Khooei A Ghiasi T Dehghan P. Pituitary glioblastoma: A case report. Iran J Cancer Prev. (2015) 8:e3436. 10.17795/ijcp-3436 26478794 PMC4606372

[24] Deng S Liu L Wang D Tong D Zhao G. Small cell glioblastoma of the Sella Turcica region: Case report and review of the literature. World Neurosurg. (2018) 110:174–9. 10.1016/j.wneu.2017.11.038 29155113

[25] Alshail E Rutka JT Becker LE Hoffman HJ. Optic chiasmatic-hypothalamic glioma. Brain Pathol. (1997) 7:799–806. 10.1111/j.1750-3639.1997.tb01065.x 9161730 PMC8098322

[26] Kornreich L Blaser S Schwarz M Shuper A Vishne TH Cohen IJ. Optic pathway glioma: Correlation of imaging findings with the presence of neurofibromatosis. Am J Neuroradiol. (2001) 22:1963–9. 11733333 PMC7973845

[27] Laws ER Weiss MH White WL. Craniopharyngioma. Skull Base. (2003) 13:55–8. 10.1055/s-2003-37554 15912160 PMC1131830

[28] Bommakanti K Panigrahi M Yarlagadda R Sundaram C Uppin MS Purohit AK. Optic chiasmatic-hypothalamic gliomas: Is tissue diagnosis essential? Neurol India. (2010) 58:833–40. 10.4103/0028-3886.73738 21150045

[29] Faggiano A Mansueto G Ferolla P Milone F de Caro MLdB Lombardi G. Diagnostic and prognostic implications of the World Health Organization classification of neuroendocrine tumors. J Endocrinol Invest. (2008) 31:216–23. 10.1007/BF03345593 18401203

[30] Sirsath NT Babu KG Das U Premlatha CS. Paranasal sinus neuroendocrine carcinoma: A case report and review of the literature. Case Rep Oncol Med. (2013) 2013:728479. 10.1155/2013/728479 23476846 PMC3586447

[31] Liu H Wang H Qi X Yu C. Primary intracranial neuroendocrine tumor: Two case reports. World J Surg Oncol. (2016) 14:138. 10.1186/s12957-016-0887-4 27138163 PMC4852410

[32] Nasi D Perano D Ghadirpour R Iaccarino C Servadei F Romano A. Primary pituitary neuroendocrine tumor: Case report and literature review. Surg Neurol Int. (2017) 8:101. 10.4103/sni.sni_450_16 28695048 PMC5473081

[33] Ibrahim M Yousef M Bohnen N Eisbruch A Parmar H. Primary carcinoid tumor of the skull base: Case report and review of the literature. J Neuroimaging. (2010) 20:390–2. 10.1111/j.1552-6569.2008.00317.x 19021842

[34] Porter DG Chakrabarty A McEvoy A Bradford R. Intracranial carcinoid without evidence of extracranial disease. Neuropathol Appl Neurobiol. (2000) 26:298–300. 10.1046/j.1365-2990.2000.00257.x 10886688

[35] Hood B Bray E Bregy A Norenberg M Weed D Morcos JJ. Primary carcinoid tumor of the cavernous sinus. World Neurosurg. (2014) 81:202.e9–13. 10.1016/j.wneu.2013.06.009 23838365

[36] Cortés Vela JJ Concepción Aramendía L Ballenilla Marco F Gallego León JI González-Spínola San Gil J. Cerebral cavernous malformations: Spectrum of neuroradiological findings. Radiologia. (2012) 54:401–9. 10.1016/j.rxeng.2011.09.004 22197483

[37] Buonaguidi R Canapicci R Mimassi N Ferdeghini M. Intrasellar cavernous hemangioma. Neurosurgery. (1984) 14:732–4. 10.1227/00006123-198406000-00014 6462408

[38] Al-Sharydah AM Al-Suhibani SS Al-Jubran SA Al-Abdulwahhab AH Al-Bar M Al-Jehani HM. Endoscopic management of Atypical sellar cavernous hemangioma: A case report and review of the literature. Int J Surg Case Rep. (2018) 42:161–4. 10.1016/j.ijscr.2017.12.006 29248833 PMC5985255

[39] Pan X Shen J Ma Y Lou H Weng Y Zhan R. Imaging characteristics of Intrasellar cavernous hemangioma: A case report. Medicine. (2020) 99:e23405. 10.1097/MD.0000000000023405 33217885 PMC7676586

[40] Al-Saiari S Al-Orabi K Farag A Brinji Z Azzouz A Mohammed T. Intrasellar cavernous hemangiomas: A case report with a comprehensive review of the literature. Surg Neurol Int. (2021) 12:58. 10.25259/SNI_622_2020 33654561 PMC7911136

[41] Kamrin RB Buchsbaum HW. Large vascular malformations of the brain not visualized by serial angiography. Arch Neurol. (1965) 13:413–20. 10.1001/archneur.1965.00470040079013 5834702

[42] Sansone ME Liwnicz BH Mandybur TI. Giant pituitary cavernous hemangioma: Case report. J Neurosurg. (1980) 53:124–6. 10.3171/jns.1980.53.1.0124 7411202

[43] Mitsuhashi T Hashimoto R Nagahama S Nagata Y. Intrasellar cavernous angioma in neurofibromatosis. Hum Pathol. (1991) 22:623–4. 10.1016/0046-8177(91)90244-J 1907595

[44] Chhang WH Khosla VK Radotra BD Kak VK. Large cavernous haemangioma of the pituitary fossa: A case report. Br J Neurosurg. (1991) 5:627–9. 10.3109/02688699109002886 1772609

[45] Lombardi D Giovanelli M de Tribolet N. Sellar and parasellar extra-axial cavernous hemangiomas. Acta Neurochir. (1994) 130:47–54. 10.1007/BF01405502 7725942

[46] Cobbs CS Wilson CB. Intrasellar cavernous hemangioma. Case report. J Neurosurg. (2001) 94:520–2. 10.3171/jns.2001.94.3.0520 11235960

[47] Chibbaro S Cebula H Ganau M Gubian A Todeschi J Lhermitte B. Multidisciplinary management of an intra-sellar cavernous hemangioma: Case report and review of the literature. J Clin Neurosci. (2018) 52:135–8. 10.1016/j.jocn.2018.03.021 29622503

[48] Jeon SCYJ Yang JH Lee I. Intrasellar cavernous hemangioma. J Korean Neurosurg Soc. (2004) 36:163–5.

[49] Zhou BY Cai BW Liu YH Fan YJ. Cavernous sinus but not intrasellar cavernous hemangioma. Neurol India. (2013) 61:442–3. 10.4103/0028-3886.117603 24005750

[50] Ma LC Li WY Chen WQ Wu YK. Intrasellar cavernous hemangioma. Neurol India. (2014) 62:95–6. 10.4103/0028-3886.128352 24608478

[51] Das S Ang LC Ramsay D. Intrasellar cavernous hemangioma presenting as pituitary adenoma: A report of two cases and review of the literature. Clin Neuropathol. (2018) 37:64–7. 10.5414/NP301012 29189199

[52] Chuang CC Jung SM Yang JT Chang CN Pai PC. Intrasellar cavernous hemangioma. J Clin Neurosci. (2006) 13:672–5. 10.1016/j.jocn.2005.08.017 16815022

[53] Poorthuis MHF Rinkel LA Lammy S Al-Shahi Salman R. Stereotactic radiosurgery for cerebral cavernous malformations: A systematic review. Neurology 11. (2019) 93:e1971–9. 10.1212/WNL.0000000000008521 31659093

[54] Saha A Tso S Rabski J Sadeghian A Cusimano MD. Machine learning applications in imaging analysis for patients with pituitary tumors: A review of the current literature and future directions. Pituitary. (2020) 23:273–93. 10.1007/s11102-019-01026-x 31907710

[55] Qiao N. A systematic review on machine learning in sellar region diseases: Quality and reporting items. Endocr Connect. (2019) 8:952–60. 10.1530/EC-19-0156 31234143 PMC6612064

